# Targeting Hypoxia-Inducible Factor-1α in Pancreatic Cancer: siRNA Delivery Using Hyaluronic Acid-Displaying Nanoparticles

**DOI:** 10.3390/pharmaceutics16101286

**Published:** 2024-09-30

**Authors:** Alice Spadea, Annalisa Tirella, Julio Manuel Rios de la Rosa, Enrique Lallana, Manal Mehibel, Brian Telfer, Nicola Tirelli, Margaret Jayne Lawrence, Kaye J. Williams, Ian J. Stratford, Marianne Ashford

**Affiliations:** 1 NorthWest Centre for Advanced Drug Delivery (NoWCADD), School of Health Science, University of Manchester, Oxford Road, Manchester M13 9PT, UK; julio.rios@inibica.es (J.M.R.d.l.R.);; 2 Max Planck Institute of Molecular Cell Biology and Genetics, Pfotenhauerstrasse 108, 01307 Dresden, Germany; 3 Division of Pharmacy and Optometry, School of Health Science, University of Manchester, Oxford Road, Manchester M13 9PT, UK; annalisa.tirella@unitn.it (A.T.); manal.mehibel@abbvie.com (M.M.); ian.j.stratford@manchester.ac.uk (I.J.S.); 4 BIOtech Research Centre, Department of Industrial Engineering, University of Trento, 38122 Trento, Italy; 5 Instituto de Investigacion e Innovacion Biomedica de Cadiz (INiBICA), Hospital Universitario Puerta del Mar, 11009 Cadiz, Spain; 6 EM Analytical Ltd., Media House, Adlington SK10 4NL, UK; 7 Precision Medicine Oncology, Abbvie Bay Area, 1000 Gateway Boulevard, South San Francisco, CA 94080, USA; 8 Laboratory of Polymers and Biomaterials, Fondazione Istituto Italiano di Tecnologia, Via Morego 30, 16163 Genova, Italy; 9 Advanced Drug Delivery, Pharmaceutical Sciences, R&D, AstraZeneca, Macclesfield M13 9PT, UK; marianne.ashford@astrazeneca.com

**Keywords:** polymeric nanoparticles, siRNA delivery, cancer, hyaluronic acid, CD44

## Abstract

**Background/Objectives:** Conventional anticancer therapies often lack specificity, targeting both cancerous and normal cells, which reduces efficacy and leads to undesired off-target effects. An additional challenge is the presence of hypoxic regions in tumors, where the Hypoxia Inducible Factor (HIF) transcriptional system drives the expression of pro-survival and drug resistance genes, leading to radio- and chemo-resistance. This study aims to explore the efficacy of targeted nanoparticle (NP)-based small interfering RNA (siRNA) therapies in downregulating these genes to enhance treatment outcomes in pancreatic cancer, a tumor type characterized by high CD44 expression and hypoxia. **Methods:** We utilized hyaluronic acid (HA)-displaying nanoparticles composed of positively charged chitosan (CS) complexed with siRNA to target and knock down HIF-1α in pancreatic cancer cells. Two NP formulations were prepared using either low molecular weight (LMW) or high molecular weight (HMW) CS. These formulations were evaluated for their internalization by cells and their effectiveness in gene silencing, both in vitro and in vivo. **Results:** The study found that the molecular weight (MW) of CS influenced the interaction between HA and CD44, as well as the release of siRNA upon internalization. The LMW CS formulation shows faster uptake kinetics, while HMW CS is more effective in gene knockdown across different cell lines in vitro. In vivo, both were able to significantly knockdown HIF-1α and some of its downstream genes. **Conclusions:** The results suggest that HMW and LMW CS-based NPs exhibit distinct characteristics, showing that both MWs have potential for targeted pancreatic cancer therapy by influencing different aspects of delivery and gene silencing, particularly in the hypoxic tumor microenvironment.

## 1. Introduction

Pancreatic cancer is one of the most lethal tumors with a 5-year survival rate of only 10%. The abundance of stromal cells and extracellular matrix, typical of this type of cancer, generate regions of low accessibility not only to nutrients and oxygen, but also to chemotherapeutics agents. The presence of hypoxia (lack of oxygen) creates an environment whereby tumor cells can adapt and modify their metabolism to survive and to become resistant to radio- and chemo-therapy [1]. This adaptation occurs through the Hypoxia Inducible Factor (HIF) transcriptional system, which regulates the expression of genes that support survival under low oxygen conditions [2]. HIF-1 is expressed by all metazoan organisms, including humans, and is constituted by two subunits, HIF-1α and HIF-1β. The HIF-1β protein subunit (also known as the aryl hydrocarbon nuclear receptor translocator, ARNT) is constitutively expressed, but the HIF-1α subunit, under oxygenated conditions, is degraded by the prolyl hydroxylase PHD2, which uses oxygen as a substrate [3]. As a result, the HIF-1α subunit accumulates in low oxygen conditions, driving the transcriptional activity of HIF-1 [4].

Several studies have highlighted the pivotal role of HIF-1α in the development and progression of pancreatic cancer, particularly in promoting treatment resistance. Overexpression of HIF-1α is linked to gemcitabine-resistant pancreatic cancer cells, and its inhibition has been shown to partially reverse epithelial-to-mesenchymal transition (EMT), a process critical to metastasis and drug resistance [5,6]. Additionally, HIF-1α is associated with lymph node metastasis and poor prognosis [7]. Thus, targeting HIF-1α is a promising therapeutic strategy for overcoming chemo-resistance and reducing metastatic potential in pancreatic cancer and other tumors [8,9,10,11]. One approach to inhibit HIF-1α activity involves RNA interference (RNAi) technology, which uses small interfering RNAs (siRNAs) of 19–21 nucleotides in length to degrade specific mRNA [12]. In the last 20 years, siRNA-based therapies have received considerable attention with the first siRNA-based nanoparticle system (patisiran, Onpattro©) being approved by the US Food Drug Administration (FDA) in 2018. With another three siRNA products approved in the following years and with multiple others currently being evaluated in clinical trials, siRNA-based agents represent an incredibly promising approach to treat a wide range of diseases including those hitherto thought to be untreatable [13].

Among non-viral carriers, positively charged polymeric nanoparticles (NPs) are frequently used for siRNA delivery because they can carry and deliver rather high doses of negatively charged siRNA, while protecting it from endonucleases [14]. Chitosan (CS) is a widely used cationic polymer for nucleic acid delivery due to its biocompatibility, low toxicity, and low immunogenicity [15,16,17]. However, an effective carrier for intracellular delivery must have specific characteristics, such as high specificity for the target site, efficient uptake by target cells, and the ability to release siRNA upon internalization. Surface modification of NPs with negatively charged molecules like hyaluronic acid (HA) can overcome the problem of unselective uptake, as well as aggregation with serum proteins [18,19]. Significantly, HA is not only the main component of the extracellular matrix, but also the main ligand of the CD44 receptor, which is overexpressed in most solid tumors, in comparison to the tissues they derive from.

CD44 is a transmembrane receptor involved in many physiological processes including cell migration, cell growth, and regulation of cell–matrix interactions. It can be present in many different isoforms due to alternative splicing where the smallest standard isoform is CD44s and variants are CD44v. The potential application of HA in tumor targeting has recently been reviewed [17,20,21]. Our prior research revealed that under conditions of equally elevated CD44 expression, the uptake of free HA in vitro was higher in cells where CD44s was the almost unique form (termed CD44s^high^) as opposed to those where CD44s was also accompanied by high expression of different CD44v; importantly, this does not necessarily indicate that CD44v binds less HA, but their presence may be used as an indicator of reduced HA uptake. Furthermore, our observations indicated that CD44s^high^ cancer cells exhibit a notably higher uptake of HA compared to CD44s^high^ fibroblasts [19].

The MW and structural properties of HA can also significantly affect its interaction with CD44, impacting binding, cellular uptake, and targeting efficiency. Higher MW HA (≥30 kDa) typically demonstrates stronger binding to CD44 than low MW HA (<30 kDa), whether it is free in solution, grafted to liposomes, or used in NPs. This is largely because longer HA chains can interact with multiple CD44 molecules simultaneously, increasing binding affinity and promoting receptor clustering, which can enhance internalization into cells [22,23].

The CD44/HA interaction has been widely investigated for the development of tumor-targeting delivery systems since the CD44 receptor is overexpressed in many cancers [24]. Here, we produce and evaluate HA-displaying CS NPs, using a 180 kDa HA, containing siRNA as an approach to achieving specificity in targeting pancreatic tumor, enhance uptake, and delivering siRNA to knockdown HIF-1α. Attention was directed to determine the internalization kinetics of two distinct NP formulations (with two different CS molecular weights (MW), 36 and 656 kDa) in two pancreatic cancer cell lines (MIA PaCa-2 and PANC-1), which are both CD44s^high^ [25]. Moreover, HIF-1α knockdown in the same cells and HIF-1α and its downstream target genes knockdown in vivo were assessed. The results of the study provide a strong foundation for considering combination studies involving gene downregulation and chemo- or radio-therapies. While previous studies have explored HA-displaying nanosystems in the treatment of other cancers or diseases [11,26,27,28,29], the specific effects of CS MW together with the use of active targeting via HA-CD44 interactions on delivery efficiency and gene knockdown in pancreatic cancer have not been previously reported, making our approach unique.

## 2. Materials and Methods

### 2.1. Chemicals and Reagents

Phosphate-buffered saline (PBS) tablets were sourced from Oxoid (Hampshire, UK). Cell culture media, L-glutamine, and a 0.25% (*w/v*) trypsin and 0.02% (*w/v*) ethylenediaminetetraacetic acid (EDTA) solution were purchased from Sigma Aldrich (Poole, Dorset, UK). Heat-inactivated fetal bovine serum (FBS) was provided by Gibco (Paisley, UK), untreated FBS by GE Hyclone (Loughborough, UK) and dimethyl sulfoxide (DMSO) by Fisher (Leicestershire, UK). Petri dishes, plates, and flasks for tissue culture were purchased from Falcon (Runcorn, UK).

### 2.2. General Cell Culture

MIA PaCa-2 and PANC-1 cancer cell lines were cultured in T75 flasks (Falcon, Runcorn, UK) using DMEM supplemented with 2 mM L-glutamine (Sigma-Aldrich, Poole, Dorset, UK) and 10% (*v/v*) heat-inactivated fetal bovine serum (FBS) (Gibco, Paisley, UK). Cells were incubated in a humidified atmosphere of 5% CO_2_ (*v/v*) at 37 °C and routinely trypsinized using a solution of 0.5% (*w/v*) trypsin and 0.2% (*w/v*) EDTA (Sigma-Aldrich, Poole, Dorset, UK).

### 2.3. Preparation of Chitosan/Hyaluronic Acid Nanoparticles

Chitosan (CS) and hyaluronic acid (HA) were used to prepare NPs [30]. Two different molecular weights (MW) of CS were used: a relatively high MW (viscosimetric average MW $\bar{M}$v = 656 kDa, HMW CS, degree of deacetylation 85%) was purchased by Sigma Aldrich UK, and a low MW ($\bar{M}$v = 36 kDa, LMW CS, degree of deacetylation 85%) was obtained by oxidative degradation of the HMW CS as previously reported [31]. HA ($\bar{M}$w = 180 kDa) was donated by Novozymes (Bagsvaerd, Denmark). To facilitate the investigation of cellular internalization, either a rhodamine B-labelled HA or a DY547-labelled siRNA (siGLO, Cyclophilin B control siRNA, target sequence: 5′-GGAAAGACUGUUCCAAAAA-3′, Dharmacon, UK) were used in the preparations of the NPs formulations. Rhodamine B-labelled HA was prepared by conjugating lissamine rhodamine B ethylenediamine (Thermo Scientific, Loughborough, UK) with HA using the procedure described by Rios et al. [32] (HA-RhoB). For the HIF-1α knockdown experiments, three different siRNAs were used, an off-target control (5′-UAAGGCUAUGAAGAGAUACUU-3′), denoted scrambled siRNA, and two different anti-HIF-1α sequences (siRNA2 5′-CUAACUGGACACAGUGUGUUU-3′ and siRNA3 5′-AGGUGGAUAUGUCUGGGUUUU-3′) (Dharmacon, UK). For in vivo experiments, anti-HIF-1α siRNA2 fluorescently labelled at the 5′ end with a Cy5^5 dye (Dharmacon, UK) was used. Preparation of NPs was undertaken in a laminar flow cabinet previously RNase-decontaminated using the RNaseZap solution (Thermo Scientific, UK). To obtain a total NP concentration of 1 mg/mL, a simple two-step methodology was followed [32]. In brief, nuclease-free distilled water (dH_2_O) was added to a 0.69 mg/mL CS solution while under magnetic agitation (1000 rpm for 10 min at room temperature) either (a) without any siRNA (preparing empty ‘siRNA free’ NPs); (b) 2.6% (*w/w*—with respect to CS) of DY547-labelled siRNA (preparing DY547-siRNA NPs for flow cytometry experiments); (c) 12.34% (*w/w*) of anti-HIF-1α siRNAs (preparing NPs for in vitro knockdown experiments); or (d) 25.86% (*w/w*) for anti-HIF-1α siRNA2 in vivo bio-distribution and knockdown experiments. The CS solution or the suspensions of siRNA/CS complex were then added into an equal volume of HA solution (1.5 mg/mL), either pristine or RhodamineB-labelled, and stirred at 1000 rpm for 30 min at room temperature to form HA/CS NPs. For in vivo experiments, the NPs obtained were concentrated by overnight dialysis using a 1 mL Float-A-Lyzer^®^G2 Dialysis Device (Spectra/Por^®^, Spectrum Lab, Thermo Scientific, Loughborough, UK) with a MW cut-off of 3.5–5 kDa. In brief, dialysis membranes were activated by soaking overnight with sterile 10% (*v/v*) ethanol solution in nuclease-free dH_2_O after which time it was washed with pure sterile nuclease-free dH_2_O. NPs (2 mL) were added and the membrane placed in a 50 mL Falcon tube previously filled with 15 mL of sterile 10% (*w/v*) 10 kDa poly(ethylene glycol) (PEG). After 12 h incubation at 4 °C, the concentrated NPs were collected, the volume recovered measured, and used for analysis and experiments. After concentration with dialysis, a typical concentration of 1.5 mg/mL NPs was recovered.

### 2.4. Nanoparticle Characterization

Dynamic light scattering (DLS) was employed to determine the hydrodynamic diameter (Z-average size), size polydispersity (PDI), and surface charge (ζ potential) of NPs. Measurements were conducted using a Zetasizer Nano ZS (model ZEN3600, Malvern Instruments Ltd., Malvern UK) equipped with a solid-state HeNe laser (λ = 633 nm) at a scattering angle of 173°. NPs were analyzed at a concentration of 1 mg/mL, in nuclease-free water at 25 °C following a 120 s pre-equilibration period. Size distributions were calculated using the method of cumulants and are reported as the mean (± standard deviation, SD) of the hydrodynamic diameter values of three independent samples. The Smoluchowski equation was used to convert the electrophoretic mobility of the samples into their ζ potential.

### 2.5. Nanoparticle Uptake in 2D Cell Culture (Flow Cytometry)

**Figure 1:**
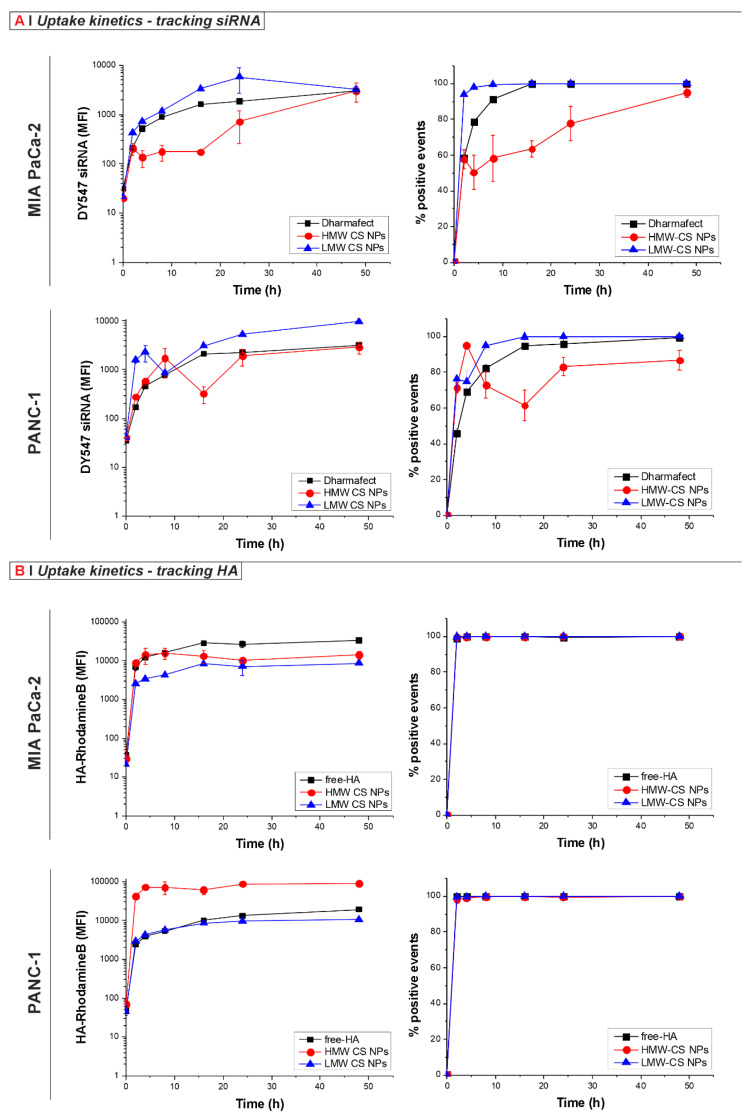
Internalization kinetics of HA-displaying NPs in MIA PaCa-2 and PANC-1 cells. (**A**) Tracking the siRNA. The cells were incubated at 37 °C for 0, 2, 4, 8, 16, 24, and 48 h with 0.125 mg/mL of LMW (blue line) or HMW (red line) CS NPs loaded with DY547-siRNA (siRNA final concentration: 40 nM; siRNA/CS weight ratio: 1.3%), or with Dharmafect transfection reagent and DY547-siRNA as a control, (black squares). Results are presented as median fluorescence intensity (MFI, **left panels**) or percentage of positive events (**right panels**). Data represent the mean (±SD) from three independent experiments. (**B**) Tracking the HA. Cells were treated at 37 °C for 0, 2, 4, 8, 16, 24, and 48 h with empty 0.125 mg/mL of LMW (blue triangles), HMW (red circles) CS (HA-RhoB) NPs, or with 0.1 mg/mL free-HA-RhoB (control, black squares), corresponding to the HA concentration in the NPs solution). Results are reported as MFI (**left panels**) or percentage of positive events (**right panels**). Data represent the mean (±SD) from three independent experiments.

MIA PaCa-2 and PANC-1 cells were seeded in 12-well plates at densities of 1 × 10^5^ and 0.5 × 10^5^ cells per well, respectively. The cells were incubated overnight at 37 °C in a 5% (*v/v*) CO_2_ atmosphere to allow attachment. Four fluorescent NP formulations prepared either with LMW or HMW CS were used at a concentration of 1 mg/mL. Before incubation, the NPs were initially diluted to 0.25 mg/mL using nuclease-free water and then further diluted to a final concentration of 0.125 mg/mL using 2X complete media (HEPES buffered, pH 7). Each well was treated with 1 mL of NPs suspension for 0, 2, 4, 8, 16, 24, and 48 h. For tracking the siRNA payload uptake, DY547-siRNA was used to prepare NPs, with DY547-siRNA complexed with Dharmacon transfectant reagent 1 (Dharmafect, GE Healthcare, Amersham UK) (40 nM final) serving as a positive transfection control. For tracking the uptake via HA, HA-RhoB was used to prepare NPs and 0.1 mg/mL (corresponding to the concentration of HA in the NPs solution) of free HA-RhoB served as a control (free-HA in Figure 1B legend). After treatment, the cells were washed twice with PBS and detached using 0.5 mL per well of 0.25/0.02 *w/v* trypsin/EDTA solution. The cells were washed twice with PBS 1× and then re-suspended in 0.5 mL of PBS 1× before flow cytometry analysis. Rhodamine B and DY547-siRNA were excited at 540/25 nm and emission was measured using a 620/40 nm filter. Untreated cells served as auto-fluorescence control and established the threshold for estimating the percentage of positive events. Data analysis was performed using the Summit 4.3 software (Dako, CO, USA).

### 2.6. Analysis of HIF-1α mRNA Downregulation

Total RNA was extracted using the RNeasy Mini Kit (Qiagen, Manchester, UK) following the manufacturer’s instructions. Cell samples were lysed with the provided buffer and stored at −80 °C until RNA extraction. DNA contamination was removed using the RNase-Free DNase Set (Qiagen, Manchester, UK). Total RNA content was quantified using a Nanodrop Lite Spectrophotometer (Thermo Scientific, UK). Reverse transcriptions of mRNA to cDNA were performed using High-Capacity cDNA Reverse Transcription Kit (Thermo Scientific, UK) following the manufacturer’s instructions using a maximum 2 μg of RNA. Quantitative PCR (qPCR) was carried out using TaqMan technology. Gene expression was analyzed using TaqMan Gene Expression Assays and TaqMan^®^ Fast Universal PCR Master Mix (2×) (Thermo Scientific, Loughborough, UK) following the manufacturer’s instructions. The genes analyzed were Vascular Endothelial Growth Factor (VEGF, Hs00173626_m1, FAM-MGB), Solute Carrier Family 2 Member 1 (GLUT-1, Hs00197884_m1, FAM-MGB), Carbonic anhydrase 9 (CA9, Hs00154208_m1, FAM-MGB), and HIF1A (HIF-1α, Hs00153153_m1, FAM-MGB). Equal amounts of cDNA were loaded for each sample within an experiment. PCR mix without cDNA served as a negative control. Endogenous controls, including Glyceraldehyde-3-phosphate dehydrogenase (GAPDH, Hs02758991_g1, VIC-MGB), β-actin (ACTB, Hs99999903_m1, VIC-MGB), and hypoxanthine phosphoribosyltransferase (HPRT, Hs99999909_m1, VIC-MGB), were used to normalize gene expression levels for relative quantitative analysis. GAPDH was used as an endogenous control for knockdown experiments under normoxia, whereas HPRT was used for hypoxia experiments since GAPDH can be hypoxia/HIF inducible in some systems. HPRT showed no expression changes relative to HIF activity. Reactions were performed in a MicroAmpTM Fast Optical 96-Well Reaction Plate (Thermo Scientific, UK) in a total reaction volume of 20 μL as per the manufacturer’s instructions. qPCR thermal cycling and data acquisition were performed in a StepOnePlus Real-Time PCR system (Thermo Scientific, UK), using the StepOne software (v2.3). The amplification conditions comprised of a holding stage (2 min at 50 °C and 10 min at 95 °C) followed by the cycling stage (40 cycles of 15 s at 95 °C and 1 min at 60 °C). The ΔΔCt method (ΔΔCt = ΔCt (target sample) − ΔCt (reference sample) = (CtD − CtB) − (CtC − CtA), B and A are housekeeping genes, and C and D genes of interest) was used to process the data and determine the relative gene expression levels between the experimental samples and controls.

### 2.7. Animal Studies

For the in vivo studies, adult female CBA nude mice, aged 8–12 weeks and bred “in house” were used. The mice were kept in a pathogen-free facility at the University of Manchester (Manchester, UK). All animal procedures were conducted under license in accordance with the UK Home Office Animals (Scientific Procedures) Act (1986) and were approved by the Animal Welfare and Ethical Review Body of the University of Manchester (Manchester, UK). Tumor-bearing mice were closely monitored daily for any changes in their overall conditions. Animal weights conform with ARRIVE guidelines. The mice were housed using individually ventilated cages with a 12-h light/dark cycle, provided with sterile food and water ad libitum, in a controlled environment for temperature and humidity. Non-aversive handling methods (cupped hand/tunnel) were employed. Each mouse and any subsequent tissue samples were identified using a unique microchip implanted identification system (Avid ID). All procedures were approved by the UK Home Office Inspectorate, the named animal care welfare officer (NACWO), and The University of Manchester Ethics Care Committee. All in vivo procedures adhered to the guidelines for the welfare and use of animals in cancer research [33].

Pancreatic MIA PaCa-2 cells were harvested during the exponential growth phase, counted, washed in serum-free media, and re-suspended in the appropriate volume of serum-free media to achieve a concentration of 2 × 10^8^ cells/mL. This cell suspension was mixed 1:1 with Matrigel and kept on ice until implantation. Each mouse received a subcutaneous injection of 0.1 mL (1 × 10^8^ cells/mL) of pre- cell/Matrigel mix at the supraspinal midline, approximately 1 cm from the base of the tail. The weight, health, and tumor volume of the mice were monitored up to three times per week, with tumor volume measurements being recorded. When the tumor volume reached about 500 mm^3^, mice were treated with NPs through intra-tumoral injection (i.tu.). NPs at a concentration of 1.5 mg/mL were mixed 1:1 with saline solution for dosing. The mice were then dosed i.tu. (2.5 mL/kg) with either scrambled siRNA or Cy5^5-anti-HIF-1α siRNA2-loaded NPs (corresponding siRNA concentration: 134 μg/kg) using an insulin/Hamilton syringe in two horizontals planes and one vertical. Mice were grouped and treated as following: Group 1 (*n* = 3): Scrambled siRNA/LMW CS NPs, Group 2 (*n* = 5): anti-HIF-1α siRNA2/LMW CS NPs, Group 3 (*n* = 3): Scrambled siRNA/HMW CS NPs, Group 4 (*n* = 5): anti-HIF-1α siRNA2/HMW CS NPs. NPs were injected at 24 h and again at 4 h before culling and harvesting the tumors, which were snap-frozen and stored at −80 °C. The HMW and LMW anti-HIF-1α siRNA2 NPs or HMW and LMW-scrambled NP-treated tumors were homogenized, RNA-extracted, and converted to cDNA, then HIF-1α and its downstream genes (GLUT1, CA9, and VEGF) expression were quantified using qPCR.

### 2.8. Statistical Analysis

The statistical analysis of the data was performed using GraphPad Prism, version 9. Details of the tests are indicated in the figure captions.

## 3. Results

### 3.1. Nanoparticle Synthesis and Characterisation

The siRNA-loaded nanoparticles (NPs) were prepared using a straightforward two-step polyelectrolyte complexation method, as previously described [31,32]. In the initial step, RNA was directly complexed by chitosan (CS), followed by coating the positively charged CS/RNA particles with hyaluronic acid (HA) in the subsequent step. For the HIF-1α knockdown studies, two different formulations were created for each CS molecular weight (MW), testing two distinct siRNA sequences—HIF-1α siRNA1 and HIF-1α siRNA2. The negative control consisted of NPs loaded with a scrambled siRNA sequence. In uptake studies, empty NPs with RhodamineB-labelled HA were employed, along with NPs with pristine HA loaded with DY547-siRNA.

The size and surface potential of the various NP formulations were evaluated using dynamic light scattering (DLS) and are detailed in Table 1. Notably, all NP formulations, irrespective of siRNA cargo, CS MW, or HA type (labelled or pristine), demonstrated consistent characteristics: a negative surface charge ranging from −25 to −42 mV (average −35 mV), Z-average hydrodynamic size of 200–300 nm, and a comparable polydispersity index (ranging from 0.13 to 0.29, averaging 0.2).

### 3.2. Nanoparticle Uptake Kinetics in Pancreatic Cancer Cells

We have previously reported that the CD44-expressing pancreatic cancer cell line MIA PaCa-2 binds and internalizes HA with high efficiency [19]. As a consequence of this observation, we wanted to investigate whether this efficient uptake of HA transferred to the efficient internalization of HA-displaying NPs. For this study, we included a second CD44^high^ pancreatic cell line, PANC1, previously studied by us for HA/CS NPs internalization and siRNA release with positive outcome [32].

The HMW and LMW CS NPs were specifically designed to allow the quantification of their uptake by tracking either their cargo (DY547-labelled siRNA) or their targeting agent (Rhodamine B-labelled HA, HA-RhoB) in both MIA PaCa-2 and PANC-1 cells, Figure 1A and Figure 1B, respectively. Of note, the NPs used for the uptake kinetics tracking HA (Figure 1B) do not contain any siRNA (Empty HA-RhoB in Table 1).

NP-treated cells were analyzed with flow cytometry at different time points of incubation. The variation in the median fluorescence intensity (MFI), shown in the left panels in Figure 1, and the % of positive events shown in the right panels in Figure 1, were used to monitor NP uptake over time. For comparison purposes, DY547-siRNA was complexed with the transfection reagent Dharmafect.

As seen in Figure 1A, when tracking the uptake of DY547-siRNA-loaded NPs in both cell lines, faster uptake kinetics were observed for the formulation prepared using LMW compared to the corresponding preparation made with HMW, as well as a generally higher MFI of the LMW CS formulation when compared to the HMW and the DY547-siRNA complexed with Dharmafect. The difference in uptake between NPs prepared using different MW of CS was even more evident when looking at the % of positive events (Figure 1A, right panel), where HMW CS NPs were not taken up by the entire population. In contrast, the uptake kinetics of LMW CS NPs better matched and exceeded the kinetics of the control Dharmafect complex in both cell lines tested, giving 100% of positive cells at early time points. The differences may be ascribed to the bigger size of the DY547-siRNA HMW CS NPs compared to the respective LMW CS formulations (Table 1), slowing down the uptake kinetics for both cell lines, as well as a different HA-representation on the NPs surface [34].

Surprisingly, different kinetics profiles were obtained when tracking the HA using NPs prepared with HA-RhoB (Figure 1B). Regardless of cell line, about 100% of cells were found to be positive after only 2 h of incubation with the NP formulations studied and with the free-HA-RhoB control. This observation is suggestive of the rapid uptake of the NPs by every cell. However, we cannot fully exclude the leakage of free Rho-HA that can also be taken up by the cells. Non-complexed HA was previously estimated to be about 20 wt % of the feed in different NP formulations [35]. In the case of the MIA PaCa-2 cells, the MFI of the cells treated with both formulations was lower when compared to the free-HA control, indicating that the NP formulations were less efficiently taken up than the free-HA control. In contrast, with the PANC-1 cells, the MFI of the HMW CS NPs was higher compared to the free-HA-RhoB control and the LMW CS NPs at every time point, which was also observed with confocal microscopy (Figure S1D) Here, the differences cannot be ascribed to the size of the NPs since both formulations were of comparable dimensions (Table 1). Instead, such contrast behavior may possibly be ascribed to a different antigen presentation on the surface of the two NP formulations and, possibly, the internalization route too.

### 3.3. Knockdown of HIF-1α in Pancreatic Cancer Cells

**Figure 2:**
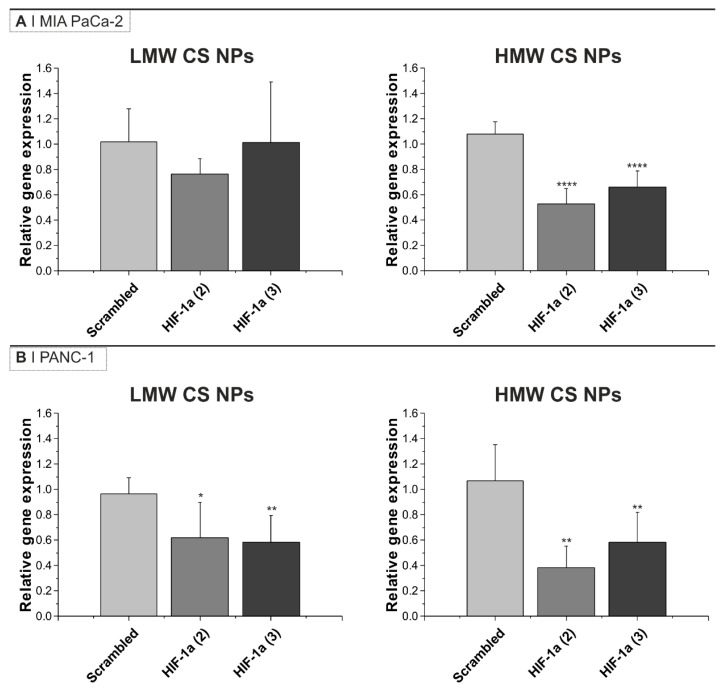
HIF-1α mRNA knockdown in (**A**) MIA PaCa-2 and (**B**) PANC-1 cells after incubation with HA-displaying NPs. Results are presented as relative gene expression using GAPDH as a loading control, with untreated cells as the reference. Cells were incubated for 24 h in normoxia with 0.125 mg/mL of either HMW or LMW CS NPs loaded with either scrambled siRNA or one of two different anti-HIF-1α sequences (siRNA final concentration = 200 nM). Data are normalized using untreated cells. Bars represent the mean ± SD from at least three independent experiments. Statistical analysis was performed using one-way ANOVA and Dunnet’s comparable test: * *p* < 0.05, ** *p* < 0.01, **** *p* < 0.0001. Transfection controls are shown in Figure S2.

Based on NP uptake results, we investigated the efficacy of the NPs in downregulating the expression of HIF-1α mRNA. To this end, MIA PaCa-2 and PANC-1 cancer cell lines were incubated with 0.125 mg/mL siRNA-loaded NPs (siRNA final concentration = 200 nM) for 24 h (Figure 2). Two different anti-HIF-1α sequences, named siRNA2 and siRNA3, were used in this study [36,37] and were firstly tested for efficacy in normoxia and hypoxia (Figure S1A,B). A scrambled siRNA was used as the negative control. Regardless of the siRNA sequence loaded in the NPs, no significant difference in size, PDI, or zeta-potential was observed (Table 1).

The cellular levels of mRNA before and after incubation with NPs were quantified via qPCR analysis. The results in Figure 2 show that the addition of anti-HIF-1α siRNA (both siRNA sequences) containing HMW CS NPs to both cell lines were able to significantly silence HIF-1α mRNA by about 50%. The addition of anti-HIF-1α siRNA containing LMW CS NPs also caused a reduction in HIF-1α mRNA expression in PANC-1 cells. On the other hand, HIF-1α mRNA silencing on MIA PaCa-2 cells with LMW CS NPs resulted in no statistical differences and a higher variability within replicates.

The difference in mRNA expression levels between the types of NPs cannot be ascribed to differences in NP size, as comparable sizes were obtained for HMW and LMW CS NPs and those containing the different siRNA sequences (Table 1). The results reported in Figure 1A suggest that, at 24 h, NP uptake in both cell line cells was higher for those prepared using LMW CS, indicating that despite being more quickly taken up and to a greater extent, the LMW-containing formulation was less efficient, possibly indicating a reduction in the amount of intact siRNA released into the cytoplasm (i.e., less efficient endosomal escape).

### 3.4. Knockdown of HIF-1α and Its Downstream Target Genes In Vivo

In vitro analysis of the pancreatic cancer cell lines treated with siRNA-loaded NPs revealed the potential role of these nanosized delivery systems as vehicles for gene silencing. However, in vitro studies alone cannot predict every aspect of the in vivo situation, e.g., presence of hypoxic, low accessible regions in solid tumors. Therefore, testing the efficacy of these NPs in pancreatic tumors in vivo was necessary.

We therefore performed intra-tumoral (i.tu.) injections of NPs into tumor-bearing mice to investigate their gene knockdown efficacy in vivo. In this regard, when the tumor volume reached about 500 mm^3^, HMW and LMW CS NPs containing either anti-HIF-1α siRNA2 or scrambled siRNA were injected twice, 24 h and 4 h before culling the mice. Tumors were harvested and the expression of HIF-1α and few of its downstream genes quantified. We selected three HIF-1α downstream genes (GLUT-1, CA9 and VEGF) since their overexpression is known to contribute to cancer progression through various mechanisms including metabolism reprogramming (for GLUT-1 and CA9) and angiogenesis (for VEGF) [2,38,39,40]. The reduction of expression of GLUT-1 following HIF-1α knockdown using LMW and HMW CS NPs was already observed by us in vitro (Figure S1C).

**Figure 3:**
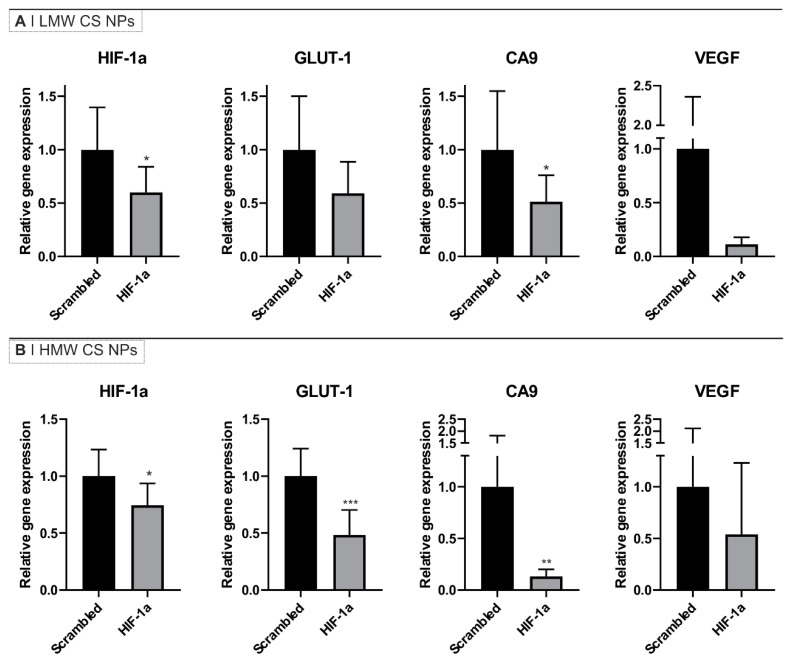
HIF-1α knockdown in MIA PaCa-2 tumors after intra-tumoral injection of HA-displaying (**A**) LMW or (**B**) HMW CS anti-HIF-1α or scrambled siRNA NPs. The results are expressed as relative gene expression using HPRT as a loading control and normalized against scrambled siRNA NPs sample. Bars represent the mean ± SD (*n* = 5). Statistical analysis was performed using unpaired *t*-test: * *p* < 0.05, ** *p* < 0.01, *** *p* < 0.001.

The results in Figure 3 show a reduction of the expression of all the genes tested, namely HIF-1α, GLUT1, CA9, and VEGF, in the tumors after exposure to NPs containing anti-HIF-1α siRNA when compared to the genes expression in tumors exposed to NPs containing scrambled siRNA. The LMW CS NPs containing anti-HIF-1α siRNA demonstrated a downregulation of about 50% of the mRNA levels of HIF-1α, GLUT-1, and CA9 and of about 90% for VEGF mRNA. However, due to variability across repeats, this was statistically significant only for HIF-1α and CA9 mRNAs (Figure 3A). The HMW CS NPs containing anti-HIF-1α siRNA showed a downregulation of about 25% for HIF-1α mRNA, 50% for GLUT-1, and 90% CA9; VEGF was also reduced to a mean of about 50%, although in this latter case, the reproducibility of the measurements were poor and as a consequence only the results for HIF-1α, GLUT-1, and CA9 genes were statistically significant (Figure 3B).

## 4. Discussion

Pancreatic cancer remains one of the most lethal cancers worldwide. Its characteristic hypoxic microenvironment, mainly governed by the action of HIF-1α, greatly contributes to the development of a malignant phenotype, which includes metabolic reprogramming, metastasis, and therapeutic resistance [41,42]. A combination therapy strategy with anti-HIF-1α siRNA delivery using NP and chemotherapy/radiotherapy would enhance the therapeutic effect of the treatment together with the possibility of reducing metastasis and relapses [11,43]. The aim of this work was to explore the use of HA-displaying NPs as an effective strategy to facilitate (i) the targeting of pancreatic cancer cells that are known to express high levels of the HA receptor CD44, (ii) the intracellular delivery of siRNA, and (iii) the knockdown of HIF-1α and its downstream target genes.

The likelihood of off-target effects of HA-displaying NPs is minimized due to several factors: pancreatic cancer cells express higher levels of CD44, while normal tissues have lower CD44 expression [25,44]. Additionally, the uptake of HA by normal cells is reported to be less efficient compared to cancer cells [19,45,46]. The siRNA targets HIF-1α, which is predominantly active in the hypoxic conditions found in solid tumors, reducing off-target effects in normal tissues [47]. Lastly, the enhanced permeability and retention (EPR) effect favors NPs accumulation in tumors [48].Two pancreatic cancer cell lines were used for the current study. The first, the MIA PaCa-2 cell line, has previously been thoroughly screened by us for its CD44 expression profile, HA binding, and internalization capacity [19]. The second, PANC-1, with a CD44 expression profile similar to MIA-PaCa-2, was previously reported by us to efficiently internalize NPs, which also resulted in a significant knockdown of cyclophilin B mRNA as a silencing target [32]. Two different MWs of CS (namely 36 and 656 kDa) were here used to prepare siRNA-loaded HA-displaying NPs using a simple two-step method [30,49]. Testing different MWs of CS was considered crucial as MW influences various characteristics of the carrier. These include stability, endosomal escape capability, interaction strength with the siRNA, the efficiency of siRNA release, and, notably, HA presentation on the NPs surface [31,50]. The choice of CS with MWs of 656 kDa and 36 kDa was guided by their distinct properties documented in the literature, as well as our previous experience with these materials [16,31,32,34,35,46,49,50,51]. HMW CS (656 kDa) was chosen for its enhanced stability and potential for improved endosomal escape, which can be crucial for drug delivery systems. Conversely, LMW CS (36 kDa) was selected for its faster cellular uptake, which can facilitate quicker therapeutic responses. The CS used for this study possessed an 84% degree of deacetylation (DD) since this level of DD was previously found to confer the greatest stability on the carrier and to favor uptake and gene silencing [31].

In general, the size of the NPs ranged from 200 to 300 nm, independent of the MWCS or whether the NPs were loaded with siRNA or not. The size distribution of the NPs was also relatively narrow with polydispersity values typically less than 0.22 (Table 1). These observations agree with those previously reported by us and other groups [31,49,51]. However, the DY547-siRNA-loaded HMW CS NPs presented a slightly higher size and dispersity than the other NPs. All the NP formulations possess a strongly negative surface charge as evidenced by their measured ζ potentials. This negative surface charge is a consequence of the HA coating, which, in addition to conferring a negative charge on the NPs, also forms a corona around the CS core [50]. The NPs prepared in the present study were obtained through a simple two-step polyelectrolyte complexation in which an excess of CS is complexed with siRNA (siRNA/CS weight ratio: 12.34%, positive to negative charge ratio 12.5), a method for which we reported a high siRNA encapsulation efficiency (about 99%) [31]. The excess of CS allows the core of the NPs to possess a positive charge, which subsequently facilitates its coating by the negatively charged HA molecules (positive to negative charge ratio 2.33).

A kinetic study was performed using flow cytometry to investigate how particle uptake in the MIA PaCa-2 and PANC-1 cell lines is affected by the macromolecular characteristics of the NPs, namely the MW of the CS used in their preparation, their siRNA loading and size, and the presentation of HA on the surface of NPs (which is known to be different between formulations). Specifically, the uptake of fluorescently labelled siRNA or HA was examined as a proxy for NP uptake. By tracking siRNA, the observation that the LMW CS NPs exhibited a faster and higher uptake rate compared to their HMW counterparts (Figure 1A) could be ascribed to the larger size of the HMW CS NPs (330 nm vs. 200 nm of the LMW CS NPs) as it has been reported that the larger the diameter of NPs, the greater the likelihood that the cells will switch from receptor-mediated endocytosis to macropinocytosis [52]. Surprisingly, when tracking the HA-RhoB (Figure 1B), the uptake kinetics did not match that recorded when tracking the nucleic acid payload. In this latter case, the HMW CS NPs were observed to be taken up to a greater extent than their LMW counterparts, while in the PANC-1 cells they were taken up to a greater extent than the free-HA control. It is important to note that when present in the NP formulations, HA differs from its soluble form in several aspects. Firstly, the NPs are not soluble in the solvent but rather are dispersed in it and so, as a consequence, the surface density of the HA CD44-binding groups on the exterior of the NPs will be different to when HA is dissolved in solvent. Specifically, HA is expected to be in a more condensed form when on the exterior surface of the NPs when compared to its soluble form as previously reported [49]. Furthermore, it cannot be excluded that PANC-1 cells might be able to uptake more efficiently large CS-rich particulates that possibly result from HA decomplexation and concomitant NP instability occurring at a physiological pH. Indeed, we have previously described the partial formation of CS aggregates (more prominently) in HMW CS NPs suspended in complete media buffered at pH 7.4 [31]. Such instability is largely abrogated when NPs are buffered at pH 6.4, as a slightly acidic pH ensures a stronger chitosan/nucleic acid/HA ionic complexation and, hence, NP stability [31]. Since the uptake experiments for the current work were performed at neutral pH, PANC-1 cells might switch to macropinocytosis for the internalization of such HMW CS aggregates, resulting in a very strong cell fluorescence. This hypothesis, however, would require further investigation.

Another factor might play a role in explaining the different extents of cellular uptake observed when tracking HA and siRNA as a proxy for NP uptake: the solution the NPs are dispersed in may contain free (i.e., non-complexed) HA. Therefore, any free-HA might result in the competitive binding with the NPs, resulting in a rapid free-HA uptake—the cells were already 100% positive within 2 h of incubation with the NPs [35].

While we did not directly compare LMW and HMW CS NPs, we found that the anti-HIF-1α HMW CS NPs resulted in a statistically significant knockdown in both cell lines, although they were internalized to a lower extent. In contrast, anti-HIF-1α LMW CS NPs achieved significant knockdown only in the PANC-1 cell line. This observation is in accordance with previous studies, which demonstrated that while increasing the MW of the CS had a negative influence on the kinetics of NP internalization, it was a positive factor for transfection, possibly due to a more ready release of siRNA from the endosomes as a result of the “proton-sponge” effect in CS [31,51]. The ability to penetrate solid tumors is another important characteristic required if a drug or other therapeutic agent is to be successfully used in cancer therapy. This is particularly relevant to the current study when the aim was to downregulate HIF-1 activity, which is elevated in the hypoxic regions of solid tumors: regions with low accessibility to blood, oxygen, and nutrients. HIF-1 is particularly important because it transcribes several genes which are involved in pathways that regulate multiple aspects of cancer biology.

Under hypoxic conditions, HIF-1 upregulates the transcription of genes encoding the glucose transporters such as GLUT-1 and GLUT-3, along with nearly all the enzymes involved in glycolysis and glycogen synthesis pathways. Additionally, HIF-1 induces the expression of CA9 which facilitates the reversible conversion of CO_2_ to carbonic acid. These proteins collectively contribute to extracellular acidification, promoting invasion and intracellular alkalization, which can support proliferation [53]. In other words, under conditions of hypoxia, HIF-1 triggers a “switch” from oxidative to a glycolytic metabolism, and thereby promotes cell survival [54,55]. HIF-1 is responsible also for the transcription of critical angiogenic growth factors, including VEGF. Once produced, these factors can bind to their receptors, which are expressed on the surface of vascular endothelial cells to promote angiogenesis and vascular remodeling, and increase the delivery of nutrients and O_2_ to the tumor [56].

For these reasons, testing the downregulation of HIF-1α mRNA in cells alone was not sufficient to assess the therapeutic capacity of the NPs. We considered it necessary to test the efficacy of NPs to deliver HIF-1α siRNA2 to solid tumors in vivo with the goal of assessing whether it was possible to downregulate HIF-1 and its downstream oncogenic genes in a complex and heterogeneous tumor microenvironment. Intra-tumoral (i.tu.) injection into mice was selected for a number of reasons, including its higher stability and efficacy at the slightly acidic pH, as it would be found in the tumor microenvironment [31].

I.tu. injection strategies have garnered considerable attention within the field of oncology as complementary therapeutic modalities aimed at enhancing local tumor control [57]. This is particularly pertinent in the context of immunotherapies, such as toll-like receptor (TLR) agonists, which are frequently administered via i.tu. routes, and some of which have advanced to clinical trials [58,59]. The advantages conferred by an i.tu. intervention are multiple. Firstly, it enables the precise, localized delivery and accumulation of the active therapeutic agent, potentially extending its influence to tumor-draining lymph nodes [60]. This targeted approach obviates the need for complex targeting strategies required to overcome biological barriers and deliver the therapeutic payload to specific tissues. Additionally, i.tu. administration enhances safety by mitigating systemic exposure, thus reducing the potential for off-target effects. Several pre-clinical and clinical studies have also demonstrated the feasibility and effectiveness of i.tu. and intra-peritoneal (i.p.) injections of siRNA in pancreatic cancer [61,62,63].

Our results demonstrated a reduction in the expression of all tested genes—HIF-1α, VEGF, GLUT-1, and CA9—following NPs administration. In particular, HMW CS formulation showed a statistically significant reduction in the expression of three out of the four genes compared to the scrambled siRNA control. Interestingly, the LMW CS formulation, which did not show a statistically significant HIF-1α knockdown in vitro with MIA-PaCa-2 cells, also exhibited a significant reduction in HIF-1α and CA9 expression in vivo upon i.tu. injection. We speculate that this higher silencing efficacy in vivo is ultimately attributed to enhanced NPs stability at the lower pH of the tumor interstitial space. It should be noted that the intra-venous (i.v.) injection of NPs was considered; however, we observed a decomplexation of the NPs via this route, whereupon the HA component of the particles almost entirely accumulated in the liver 48 h after injection whereas the siRNA payload (most likely together with CS) was found mainly in the kidneys, as it has been reported also by others [64] (Figures S2–S6).

## 5. Conclusions

Our results highlighted the efficacy of anti-HIF-1α siRNA2-loaded NPs for gene knockdown both in vitro and in vivo, proving their potential in tumor targeting and for their use in combination therapy with anti-tumoral drugs and/or radiotherapy. This approach presents a promising strategy for addressing hypoxia-driven treatment resistance in pancreatic cancer by downregulating HIF-1α, a key regulator of tumor survival under low oxygen conditions. By enhancing the sensitivity of cancer cells to chemotherapy and radiotherapy, the siRNA-loaded NPs could potentially reduce metastasis and improve treatment outcomes. The selective targeting of the CD44 receptor with HA-displaying NPs further ensures cancer-specific uptake, minimizing off-target effects and potentially improving clinical outcomes for patients. However, some challenges are still associated to this approach. These include ensuring the stability and efficiency of NPs during systemic delivery, as our study used i.tu. injections, which bypass challenges such as NPs de-complexation, immune clearance, and distribution throughout the body. Additionally, long-term studies are required to assess the persistence of gene knockdown effects and to investigate the potential for tumors to develop resistance to HIF-1α inhibition over time. Overcoming these challenges will be essential for realizing the full clinical potential of anti-HIF-1α siRNA-loaded NPs in treating pancreatic cancer and other hypoxia-driven malignancies.

## Figures and Tables

**Table 1:** Physico-chemical characteristics of high molecular weight (HMW) and low molecular weight (LMW) chitosan (CS) HA-displaying nanoparticle (NP) formulations used in the study.

	Z-Average Hydrodynamic Size (nm) ± SD	PDI ± SD	ζ Potential (mV) ± SD
HMW CS NPs			
Empty (HA-RhoB)	236 ± 23	0.21 ± 0.01	−25 ± 1
Scrambled siRNA	261 ± 38	0.17 ± 0.03	−42 ± 7
HIF-1α siRNA2	276 ± 40	0.16 ± 0.02	−38 ± 1
HIF-1α siRNA3	229 ± 5	0.13 ± 0.02	−36 ± 1
DY547-siRNA	331 ± 23	0.29 ± 0.02	−35 ± 1
LMW CS NPs			
Empty (HA-RhoB)	216 ± 37	0.22 ± 0.04	−34 ± 1
Scrambled siRNA	287 ± 46	0.16 ± 0.04	−39 ± 4
α-HIF-1 siRNA2	280 ± 58	0.17 ± 0.03	−39 ± 1
α-HIF-1 siRNA3	235 ± 10	0.19 ± 0.03	−38 ± 1
DY547-siRNA	207 ± 32	0.22 ± 0.01	−39 ± 1

## Data Availability

The original contributions presented in the study are included in the article/Supplementary Material, further inquiries can be directed to the corresponding author.

## Supplementary Materials

The following supporting information can be downloaded at https://www.mdpi.com/article/10.3390/pharmaceutics16101286/s1: Figure S1: HIF-1α mRNA levels in normoxia after HIF-1α mRNA knockdown in MIA PaCa-2 cells. HIF-1α and GLUT-1 mRNA levels in hypoxia after HIF-1α mRNA knockdown in MIA PaCa-2 cells. HIF-1α and its downstream target gene GLUT-1 mRNA levels in MIA PaCa-2 cells after incubation with NPs; Confocal microscopy imaging of PANC-1 cells treated for 24 h with HMW and LMS CS NPs. Figure S2: Whole body imaging of control group xenografts. Figure S3: Organs and tumor imaging of control group xenografts. Figure S4: Whole body imaging of LMW CS/HA NPs-treated xenografts. Figure S5: Organs and tumors imaging of LMW CS/HA NPs-treated MIA PaCa-2 xenografts. Figure S6: Organs and tumors imaging of LMW CS/HA NPs-treated PANC-1 xenografts.
