# Supplementary material for: Targeting Hypoxia-Inducible Factor-1α in Pancreatic Cancer: siRNA Delivery Using Hyaluronic Acid-Displaying Nanoparticles
Source: Pharmaceutics. 2024 Sep 30;16(10):1286. doi: 10.3390/pharmaceutics16101286 (PMC11510765; doi:10.3390/pharmaceutics16101286)
Supplement: Supplementary file 1 [file pharmaceutics-16-01286-s001.zip › pharmaceutics-3191878-supplementary.pdf]

pharmaceutics

Supporting Information

Targeting Hypoxia-Inducible Factor-1α in Pancreatic Cancer: siRNA Delivery Using Hyaluronic Acid-Displaying Nanoparticles

Alice Spadea [1,2,3,*], Annalisa Tirella [3,4], Julio Manuel Rios de la Rosa [1,3,5], Enrique Lallana [1,3,6], Manal Mehibel [3,7], Brian Telfer [3], Nicola Tirelli [3,8], Margaret Jayne Lawrence [1,3], Kaye J. Williams [3], Ian J. Stratford [3] and Marianne Ashford [9]

1 NorthWest Centre for Advanced Drug Delivery (NoWCADD), School of Health Science, Unversity of Manchester, Oxford Road, Manchester, UK
2 Max Planck Institute of Molecular Cell Biology and Genetics, Pfotenhauerstrasse 108, 01307 Dresden, Germany
3 Division of Pharmacy and Optometry, School of Health Science, University of Manchester, Oxford Road, Manchester, UK
4 BIOtech Research Centre, Department of Industrial Engineering, University of Trento, Trento, Italy
5 Instituto de Investigacion e Innovacion Biomedica de Cadiz (INiBICA), Hospital Universitario Puerta del Mar, Cadiz, Spain
6 EM Analytical Ltd., Media House, Adlington, UK
7 Precision Medicine Oncology, Abbvie Bay Area, 1000 Gateway Boulevard, South San Francisco, CA 94080, USA
8 Laboratory of Polymers and Biomaterials, Fondazione Istituto Italiano di Tecnologia, Via Morego 30, 16163 Genova, Italy
9 Advanced Drug Delivery, Pharmaceutical Sciences, R&D, AstraZeneca, Macclesfield, UK;
* Correspondence: spadea@mpi-cbg.de

Hypoxic genes expression and knockdown investigation

It was necessary to test the efficacy of the anti-HIF-1α siRNA transfection in normoxic and in hypoxic conditions using a commercially available transfection reagent (Dharmafect, Dharmacon, UK). Firstly, we tested the knockdown of HIF-1α mRNA using qPCR in normoxia using two different siRNAs (namely siRNA2 and siRNA3) and two different concentrations of starting cDNA (25 and 50 ng) (Figure S1A). The results are showing about 95% knockdown for siRNA2 and >80% knockdown for siRNA3. Then, we tested the expression and the downregulation of HIF-1α and one of its downstream target genes (GLUT-1) after transfection of the sequence siRNA2 in hypoxia. Hypoxic conditions were generated using a Whitley H35 Hypoxystation (Don Whitley Scientific Limited, Shipley, UK) maintained at 1% O_2.

Briefly, cells were initially incubated for 16 hours in normoxia with an anti-HIF-1α siRNA in transfection media, then cells were transferred in hypoxia (without removing the transfection media) and lysed at different time points for RNA extraction and qPCR analysis. Results reported in figure S1B showed that to a HIF-1α knockdown between 50-70% corresponded a reduction of GLUT-1 mRNA already after 2 hours (25% reduction compared to the scrambled control), although the maximum reduction was obtained after 24 hours incubation in hypoxia and a total of 40 hours incubation with anti-HIF-1α siRNA (57% GLUT-1 knockdown from a 72% HIF-1α knockdown).

Knowing that the best knockdown was obtained after 24 hours exposure to hypoxia and the siRNA was effective already after 16 hours incubation (see the 66% HIF-1α knockdown at 0h in figure S1B), the protocol adopted for the NPs treatment was the following: cells were initially incubated in hypoxia for 6 hours to increase the levels of GLUT-1 for further knockdown and to create an environment comparable to what it will be encountered *in vivo*, where NPs will have to knockdown genes in hypoxic area of the tumors. Subsequently, anti-HIF-1α siRNA-loaded (sequence siRNA2) or scrambled siRNA-loaded

LMW or HMW CS NPs were added maintaining cells in hypoxia for additional 24 hours. Cells were then lysed and the RNA extracted for qPCR analysis. Results reported in figure S1C showed that, firstly, hypoxia successfully induced the transcription of GLUT-1 mRNA while, as expected, no increase of HIF-1α mRNA levels were observed. Anti-HIF-1α siRNA-loaded LWM CS NPs were able to knockdown 22% of HIF-1α compared to the scrambled control, while the HMW were slightly more efficient with 33% HIF-1α knockdown. Interestingly, this was translated to a knockdown of GLUT-1, in particular, anti-HIF-1α siRNA-loaded LWM CS NPs silenced 20% of the hypoxia induced fraction of GLUT-1 and the anti-HIF-1α siRNA-loaded HWM CS NPs silenced 34% of the hypoxia induced GLUT-1, thus resulting, also for GLUT-1, more efficient than the LMW CS NP counterpart.

The preliminary imaging study reported in panel D revealed that HMW CS (blue) is more visible and appears complexed with siRNA (red) and HA (green) at the plasma membrane, where the three components are co-localized. This confirms the HMW CS higher affinity for siRNA compared to LMW CS. A strong HA (green) signal was detected in cells treated with HMW CS NPs compared to LMW CS NPs. This observation aligns with the flow cytometry data, although we cannot exclude the possibility that membrane-bound components are lost during trypsinization, as trypsin can remove membrane-bound HA [1]. Notably, in the knockdown experiments (also conducted at 24 hours), no trypsin was used, suggesting that no material was lost in those cases. Both formulations show uncomplexed siRNA (red) inside the cells, likely localized in organelles such as endosomes, which appear as puncta in the images. Although it's no possible to quantify this due to lack replicates, it seems that the LMW formulation contains higher levels of uncomplexed siRNA. LMW CS is less visible but appears fully decomplexed, which might account for the higher intracellular siRNA observed with this formulation.

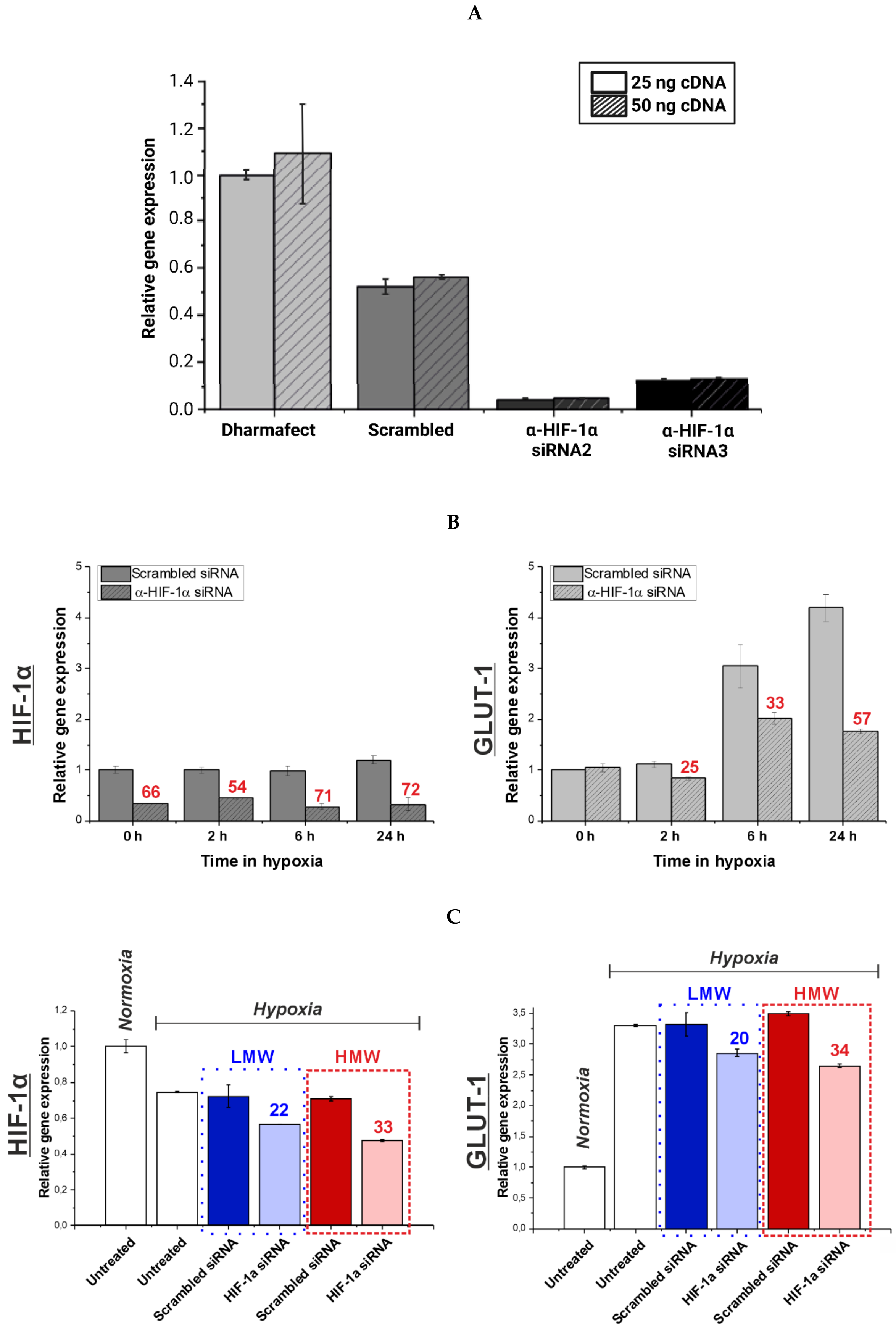
A
25 ng cDNA
50 ng cDNA
Relative gene expression
1.4
1.2
1.0
0.8
0.6
0.4
0.2
0.0
Dharmafect
Scrambled
α-HIF-1α siRNA2
α-HIF-1α siRNA3
B
HIF-1α
Relative gene expression
Scrambled siRNA
α-HIF-1α siRNA
66
54
71
72
0 h
2 h
6 h
24 h
Time in hypoxia
GLUT-1
Relative gene expression
Scrambled siRNA
α-HIF-1α siRNA
25
33
57
0 h
2 h
6 h
24 h
Time in hypoxia
C
HIF-1α
Relative gene expression
Normoxia
Hypoxia
LMW
HMW
22
33
Untreated
Untreated
Scrambled siRNA
HIF-1a siRNA
Scrambled siRNA
HIF-1a siRNA
GLUT-1
Relative gene expression
Normoxia
Hypoxia
LMW
HMW
20
34
Untreated
Untreated
Scrambled siRNA
HIF-1a siRNA
Scrambled siRNA
HIF-1a siRNA

D

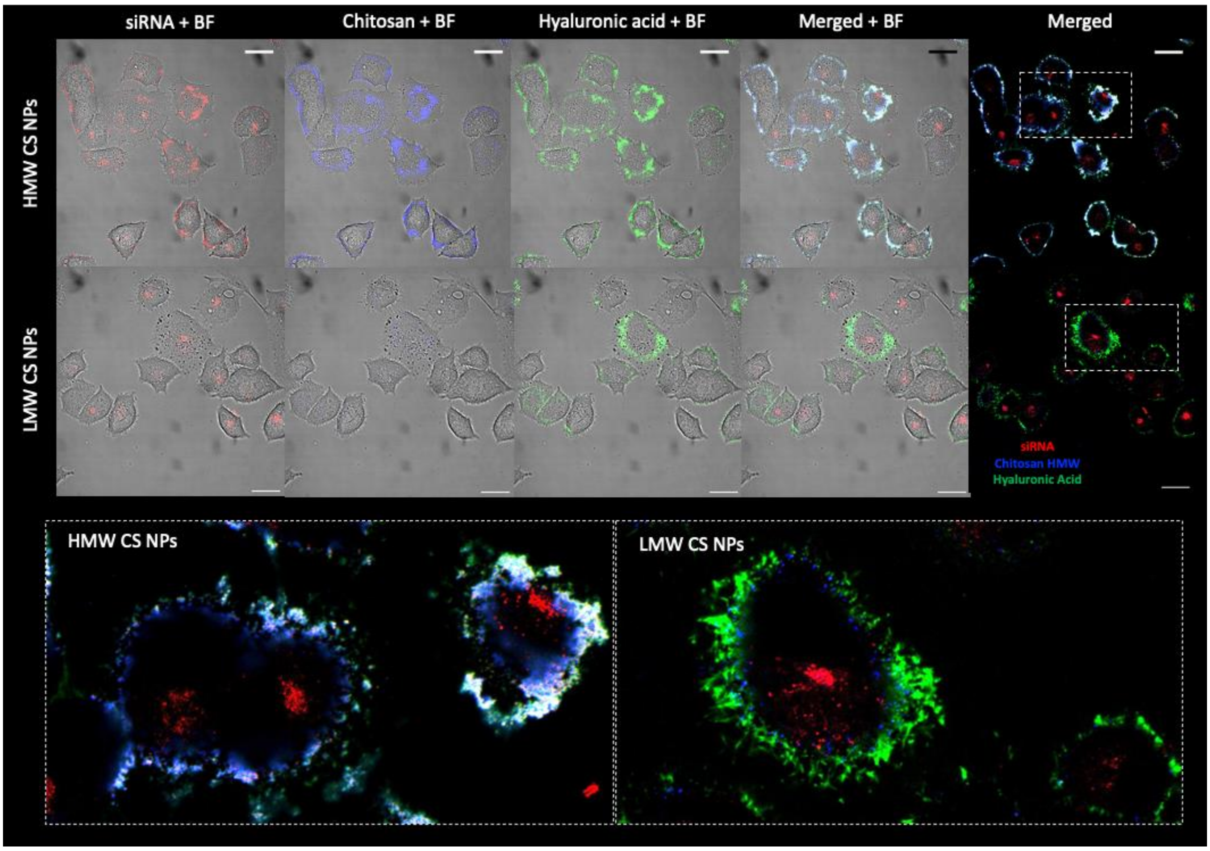

Figure S1. (A) HIF-1α mRNA levels in normoxia after HIF-1α mRNA knockdown in MIA PaCa-2 cells. The siRNA transfection was performed using anti-HIF-1α siRNA2 or anti-HIF-1α siRNA3 with Dharmafect reagent 1 following manufacturer's instructions. Cells were incubated for 24 hours in normoxia with the siRNA in transfection media (siRNA concentration = 25 nM final), then lysed for RNA extraction and cDNA production and qPCR analysis was performed testing two different concentrations of starting cDNA (25 and 50 ng). Cells transfected with a scrambled siRNA and cells incubated with the only Dharmacon reagent were used as a control. The results are expressed as relative gene expression using HPRT as a loading control. Values are the mean (± SD) of triplicates within one single experiment (n=1). **(B)** HIF-1α and GLUT-1 mRNA levels in hypoxia after HIF-1α mRNA knockdown in MIA PaCa-2 cells. The siRNA transfection was performed using anti-HIF-1α siRNA2 and Dharmafect following manufacturer's instructions. Cells were pre-incubated for 16 hours in normoxia with the siRNA in transfection media (siRNA concentration = 25 nM final), then transferred in hypoxia and lysed after 0, 2, 6 or 24 hours for RNA extraction and qPCR analysis. Cells transfected with a scrambled siRNA were used as a control. The results are expressed as relative gene expression using HPRT as a loading control. Values are the mean (± SD) of triplicates within one single experiment (n=1). The numbers in red on top of the columns represent the percentage of knockdown compare to the scrambled siRNA control at the corresponding time point. **(C)** HIF-1α and its downstream target gene GLUT-1 mRNA levels in MIA PaCa-2 cells after incubation with NPs. The experiments were performed in media HEPES buffered, pH = 7-7.4. The results are expressed as relative gene expression using HPRT as a loading control and untreated cells as reference. Cells were incubated in hypoxia for 6 hours then LMW (light blue columns) or HMW (light red columns) CS NPs loaded with anti-HIF-1α siRNA2 (siRNA concentration = 200 nM final) were added for other 24 hour in hypoxia. Untreated or cells incubated NPs loaded with a scrambled siRNA-loaded NPs (dark blue and dark red columns) were used as negative controls. Untreated cells kept in normoxia were also included as a control. Values are the mean (± SD) of triplicates within one single experiment (n=1). The numbers on top of the HIF-1α siRNA columns represent the percentage of knockdown respect to the Scrambles siRNA controls in hypoxia for HIF-1α gene and the induction from normoxia to hypoxia for GLUT-1 gene. **(D)** PANC-1 cells were treated with HMW and LMW CS HA-displaying NPs for 24 hours. The NPs were triple-labeled, with CS shown

in blue (CS-Alexa405), HA in green (HA-FITC), and siRNA in red (siRNA-DY547). The top panels report each channel ovelayed with brightfield allowing for clear visualization of the cell membrane. The final panels on the right display an overlay of all three channels withoutbrightfield. The bottom panels are a magnification of the top right panels as indicated. Scale bars = 25 µm.

Nanoparticles de-complex after intra-venous injection in tumour bearing mice

Mice of groups 2 and 4 were treated with Cy5^5-siRNA/LMW CS/HA-RhoB loaded NPs via an i.v. injection. Two days after injection mice were anesthetised and scanned using a PhotonIMAGER™ instrument (Biospace Lab, France). Two different filter settings were used to detect the Rhodamine B (ex. 545 nm, em. 567 nm) bound to the HA and the Cy5^5 dye (ex. 682 nm, em. 710 nm) bound to 5′ end of the siRNA, both present at the same time in the carrier.

Firstly, untreated control groups 1 and 3 were scanned in order to investigate whether any background signal was detectable. Figure S6 reports whole body imaging of the control mice and no fluorescence was observed using the Rhodamine B filter. This was expected since control mice were not treated with any fluorescently labelled moieties. However, surprisingly, fluorescence was detected using the Cy5^5 filter.

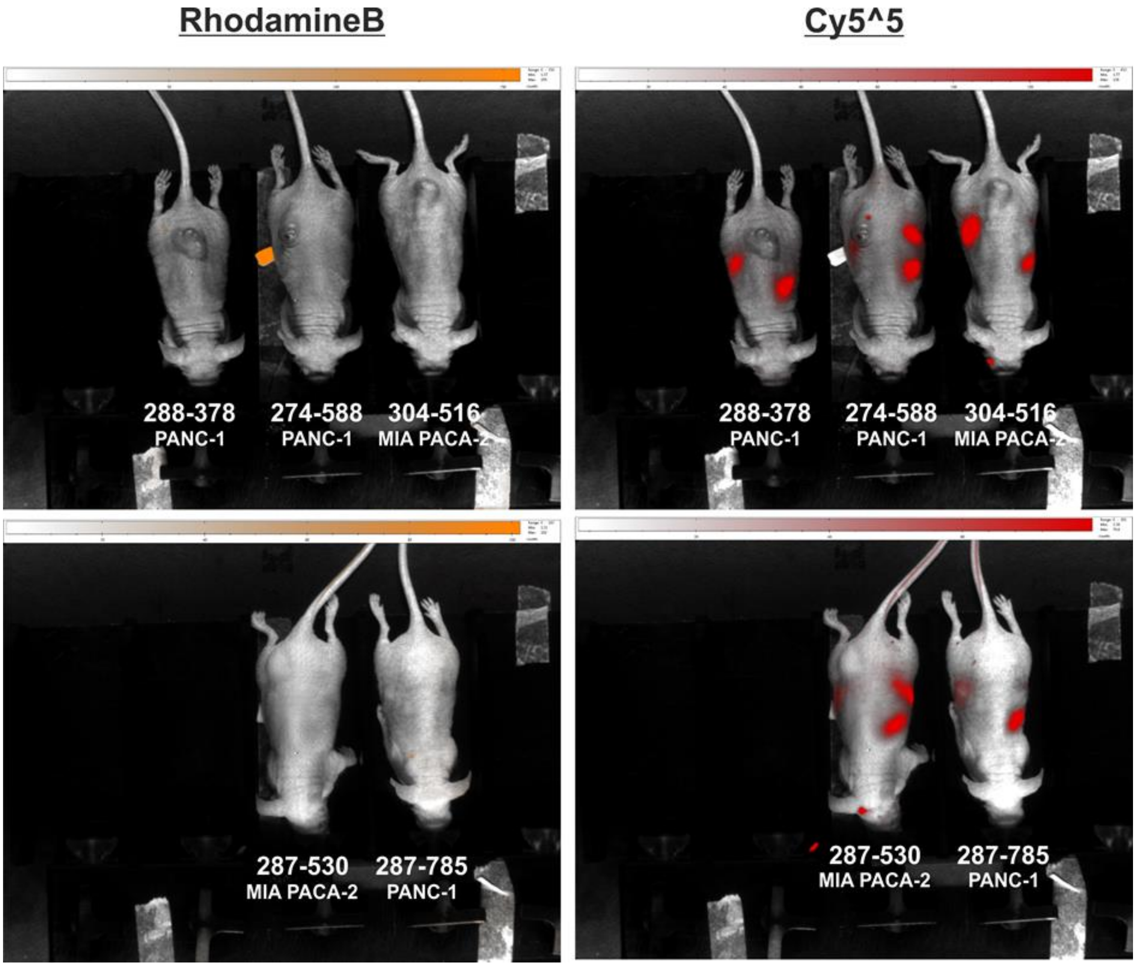

Figure S2. Whole body imaging of control group xenografts. Mice were grouped as described in section 2.11.4. Group 1 and group 3 were anesthetised and scanned with a PhotonIMAGER™ instrument for fluorescence using the Rhodamine B filter for the detection of HA-RhoB (left panels) followed by the Cy5^5 filter for the detection of siRNA(right panels) within the NPs.

In order to have a clearer idea on why and which organs emitted fluorescence in the near-infrared observed after the whole body scan (figure S6), the organs and the tumour from mouse 287-530, randomly chosen as an example, were excised and scanned a second

time for emission detection measurements. From figure S7 a clear fluorescence using the Cy5^5 filter was detected in the stomach and in the food used to feed mice. No fluorescence was, instead, detected using the Rhodamine B filter.

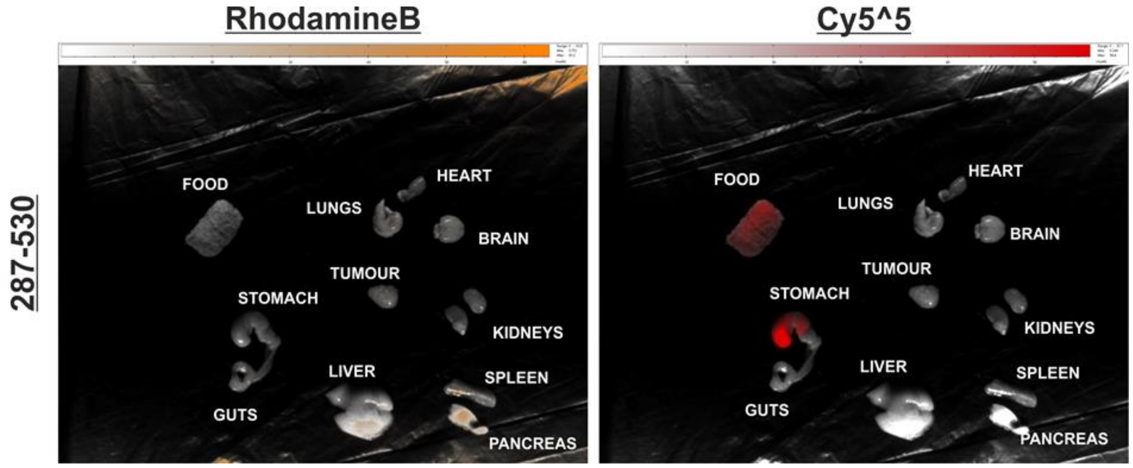

Figure S3. Organs and tumour imaging of control group xenografts. 287-530 mouse from group 1 was anesthetised, culled and the organs and the tumour excised and scanned with a PhotonIMAGER™ instrument for fluorescence using the Rhodamine B filter for the detection of HA-RhoB (left panels) followed by the Cy5^5 filter for the detection of siRNA (right panels) within the NPs. A piece of mice food was also added to the analysis.

Then mice from groups 2 and 4 were subjected to the total body scan process using the same filters as described for the controls. Figure S8 showed that HA (Rhodamine B filter) was more detectable in MIA PaCa-2 xenografts than in PANC-1 where the most of fluorescence seemed to have remained in the tail (Avid ID: 286-071 and 283-830). This was also seen in the MIA PaCa-2 xenograft 300-376, however, a strong detectable fluorescence was also measurable in the tumour area. Cy5^5 signal from the siRNA was detected in areas comparable to the controls seen in figure S6 and also in other areas including the tail in mouse 300-376 (MIA PaCa-2 xenograft) and in PANC-1 xenografts was detected in the tumour area as well as in the tail.

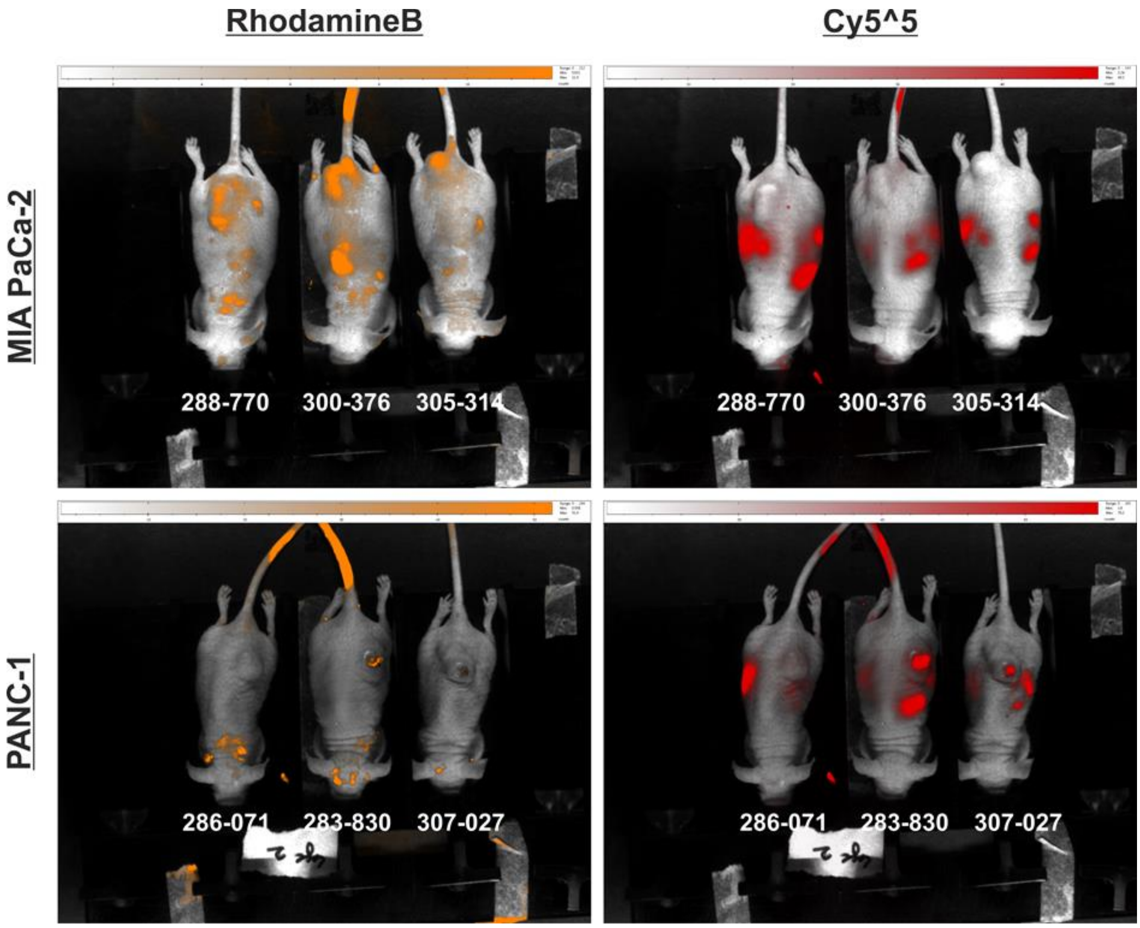

Figure S4. Whole body imaging of LMW CS/HA NPs treated xenografts. Mice were grouped as described in section 2.11.4. Group 2 (top panels, MIA PaCa-2 xenografts LMW CS NPs treated) and group 4 (bottom panels, PANC-1 xenografts LMW CS NPs treated) were anesthetised and scanned with a PhotonIMAGER™ instrument for fluorescence using the Rhodamine B filter for the detection of HA-RhoB (left panels) followed by the Cy5^5 filter for the detection of siRNA(right panels) within the NPs.

Mice bearing MIA PaCa-2 xenografts were then culled and tissues excised for a second scan to analyse the organs distribution of fluorescent NPs. The fluorescence detected initially in the stomachs of the controls resulted in their subsequent omission from the excised tissues of the secondary scans (of the internal organs) due to the realisation that the feedstuffs used for the mice produced high fluorescence levels detectable using the Cy5^5 filter. Figure S9 showed that HA, was strongly detected in the liver with lesser presence detected in the lungs and tumour. The siRNA produced a very high level of measurable fluorescence in the kidneys and to a lesser extent in the pancreas, spleen and the liver. A similar situation was observed in the PANC-1 xenografts (figure S10) where HA produced a high fluorescence level in the liver with lesser fluorescence detection in the kidneys (Avid ID: 286-071). The siRNA, again, accumulated in the kidneys and pancreas. Our results are in line with other reports on formulations with similar compositions. Generally, free HA and HA-based nanosystems are known to accumulate primarily in the liver, followed by the spleen, tumor (for tumor-bearing mice), and kidneys after intravenous (i.v.) injection [2, 3]. This pattern is consistent with HA's known excretion pathway through the liver. In contrast, siRNA is primarily found to accumulate in the kidneys, with some distribution in the intestines and liver [4]. This is expected as siRNA molecules are typically cleared from the bloodstream through renal filtration. CS-based NPs have also been shown to accumulate predominantly in the kidneys [5-7]. Overall, these studies

suggest that the biodistribution of HA and chitosan-based formulations largely follows known clearance pathways, with predominant accumulation in the liver and kidneys.

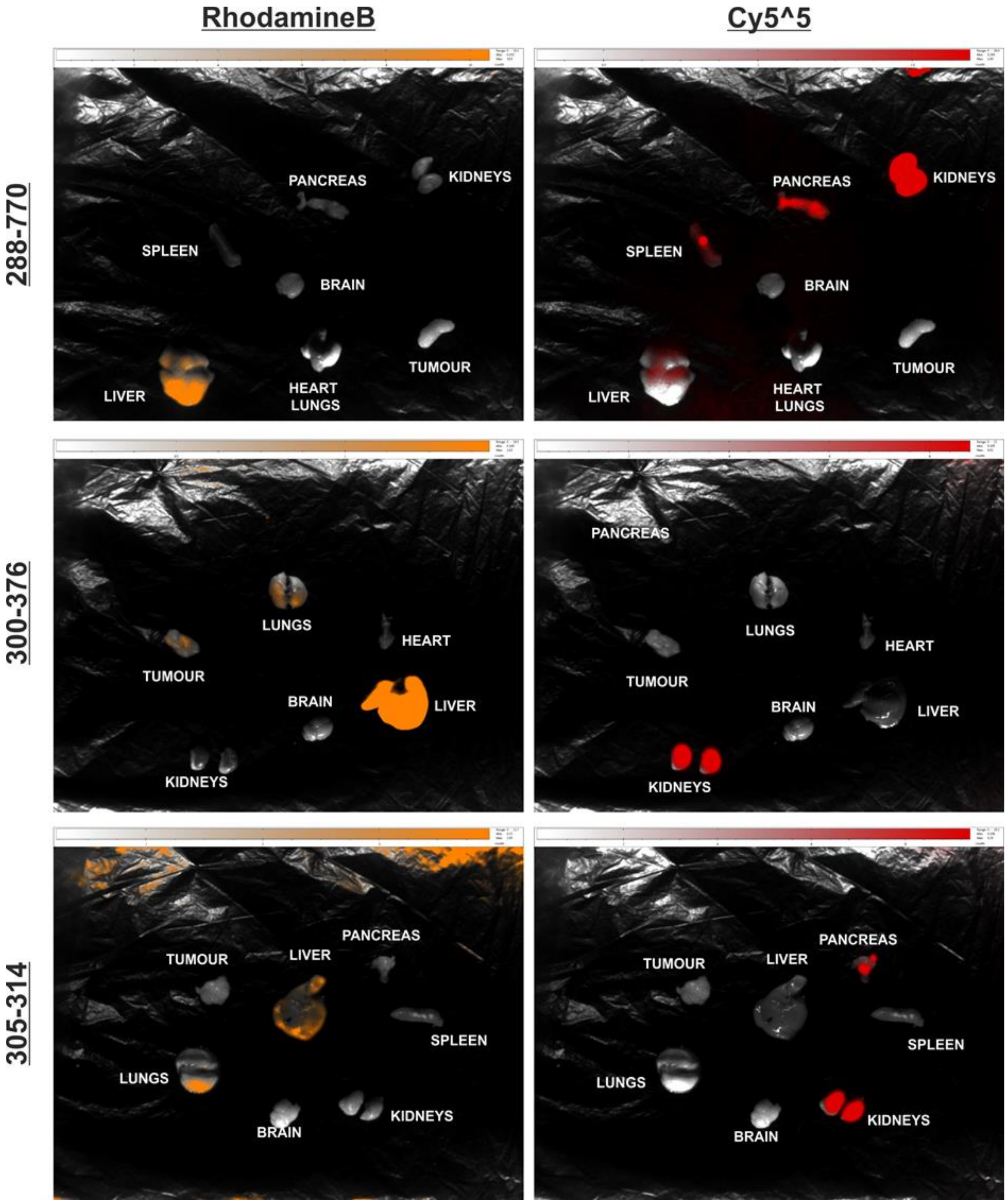

Figure S5. Organs and tumours imaging of LMW CS/HA NPs treated MIA PaCa-2 xenografts. 288-770, 300-376, 305-314 mice from group 2 were anesthetised, culled and organs and the tumour excised and scanned with a PhotonIMAGER™ instrument for fluorescence using the Rhodamine B filter for the detection of HA-RhoB (left panels) followed by the Cy5^5 filter for the detection of siRNA (right panels) within the NPs.

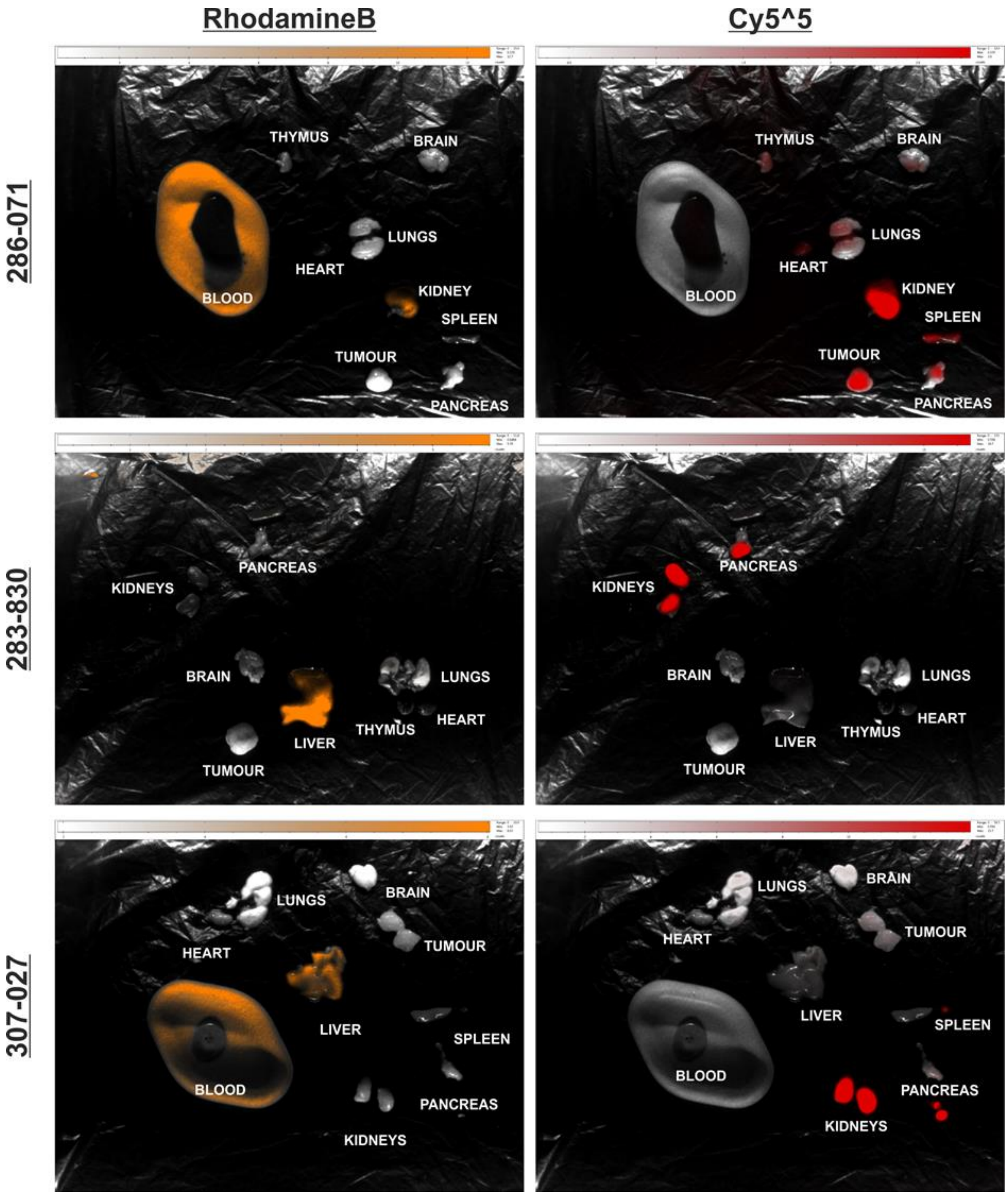

Figure S6. Organs and tumours imaging of LMW CS/HA NPs treated PANC-1 xenografts. 286-071, 283-830, 307-027 mice from group 4 were anesthetised, culled and organs and the tumour excised and scanned with a PhotonIMAGER™ instrument for fluorescence using the Rhodamine B filter for the detection of HA-RhoB (left panels) followed by the Cy5^5 filter for the detection of siRNA(right panels) within the NPs.

Material and Methods

Intra-tumoural injection of nanoparticles

Mice were then dosed i.v. (10 mL/kg) with siRNA/LMW CS/HA-RhoB NPs (corresponding siRNA concentration: 333 µg/kg).
Mice were grouped and treated as following:
Group 1 (n=3): MIA PaCa-2 cells, untreated
Group 2 (n=3): MIA PaCa-2 cells, siRNA/LMW CS/HA-RhoB NPs
Group 3 (n=3): PANC-1 cells, untreated
Group 4 (n=3): PANC-1 cells, siRNA/LMW CS/HA-RhoB NPs

48 hours post NPs injection, mice were transported to the Wolfson Imaging Centre (WMIC) for fluorescence scanning (figure 2.2). Mice were injected with 0.2 mL/mouse of pimonidazole hydrochloride (Hypoxyprobe) intraperitoneally (i.p.) two hours before being anaesthetised by placing in an induction chamber and turning the isoflurane to 2% (oxygen at 2 L/min). Pimonidazole hydrochloride is a 2-nitroimidazole compound that forms adducts in cells with an oxygen tension of 10 mmHg or less at 37°C. Pimonidazole hydrochloride was prepared in saline (0.9% w/v) at 10 mg/mL. Mice were then transferred to the heated imaging bed/holder and maintained under anaesthesia for the duration of the scan. The fluorescence signal for the whole body of each mouse from the siRNA (cargo, Cy5^5) and from the HA (carrier, Rhodamine B) were recorded in two differential scan of 5 minutes exposure each using an approved designed rodent fluorescence PhotonIMAGER™ instrument (Biospace Lab, France) and a Photo-Acquisition software analysis package. Background images (5 minutes exposure) were automatically acquired by the software and used to normalise the fluorescence signals. Image analysis was carried out using M3Vision™ software. After a first scan of whole body, mice were euthanised by an approved method. Tumours and organs were excised and scanned post removal from the sacrificed mouse (30 second fluorescence detection program and then a 30 seconds background measurement).

References

1. Spadea, A., et al., *Evaluating the Efficiency of Hyaluronic Acid for Tumor Targeting via CD44.* Mol Pharm, 2019. **16**(6): p. 2481-2493.
2. Ganesh, S., et al., *In vivo biodistribution of siRNA and cisplatin administered using CD44-targeted hyaluronic acid nanoparticles.* J Control Release, 2013. **172**(3): p. 699-706.
3. Thomas, R.G., et al., *Paclitaxel loaded hyaluronic acid nanoparticles for targeted cancer therapy: in vitro and in vivo analysis.* Int J Biol Macromol, 2015. **72**: p. 510-8.
4. Huang, Y., et al., *Elimination pathways of systemically delivered siRNA.* Mol Ther, 2011. **19**(2): p. 381-5.
5. Gao, S., et al., *The effect of chemical modification and nanoparticle formulation on stability and biodistribution of siRNA in mice.* Mol Ther, 2009. **17**(7): p. 1225-33.
6. Hyung Park, J., et al., *Self-assembled nanoparticles based on glycol chitosan bearing hydrophobic moieties as carriers for doxorubicin: in vivo biodistribution and anti-tumor activity.* Biomaterials, 2006. **27**(1): p. 119-26.
7. Alameh, M., et al., *siRNA Delivery with Chitosan: Influence of Chitosan Molecular Weight, Degree of Deacetylation, and Amine to Phosphate Ratio on in Vitro Silencing Efficiency, Hemocompatibility, Biodistribution, and in Vivo Efficacy.* Biomacromolecules, 2018. **19**(1): p. 112-131.
